# DNMT1 mediates metabolic reprogramming induced by Epstein–Barr virus latent membrane protein 1 and reversed by grifolin in nasopharyngeal carcinoma

**DOI:** 10.1038/s41419-018-0662-2

**Published:** 2018-05-23

**Authors:** Xiangjian Luo, Liping Hong, Can Cheng, Namei Li, Xu Zhao, Feng Shi, Jikai Liu, Jia Fan, Jian Zhou, Ann M. Bode, Ya Cao

**Affiliations:** 1 Key Laboratory of Carcinogenesis and Invasion, Chinese Ministry of Education, Xiangya Hospital, Central South University, Hunan 410078 Changsha, China; 2 Cancer Research Institute, School of Basic Medicine, Central South University, Hunan 410078 Changsha, China; 3 Key Laboratory of Carcinogenesis, Chinese Ministry of Health, Hunan 410078 Changsha, China; 4 School of Pharmacy, South-Central University for Nationalities, Hubei 430074 Wuhan, China; 5 The Zhongshan Hospital Shanghai Medical School, Fudan University, Key Laboratory for Carcinogenesis and Cancer Invasion, Chinese Ministry of Education, 200000 Shanghai, China; 6 The Hormel Institute, University of Minnesota, Austin, MN 55912 USA; 7 Research Center for Technologies of Nucleic Acid-Based Diagnostics of Infectious Diseases and Cancer, Hunan, Changsha China; 8 National Joint Engineering Research Center for Genetic Diagnostics and Therapeutics, Hunan Changsha, China

## Abstract

Cancer cells frequently adapt fundamentally altered metabolism to support tumorigenicity and malignancy. Epigenetic and metabolic networks are closely interactive, in which DNA methyltransferases (DNMTs) play important roles. Epstein–Barr virus (EBV)-encoded latent membrane protein 1 (EBV-LMP1) is closely associated with nasopharyngeal carcinoma (NPC) pathogenesis because it can trigger multiple cell signaling pathways that promote cell transformation, proliferation, immune escape, invasiveness, epigenetic modification, and metabolic reprogramming. Our current findings reveal for the first time that LMP1 not only upregulates DNMT1 expression and activity, but also promotes its mitochondrial translocation. This induces epigenetic silencing of *pten* and activation of AKT signaling as well as hypermethylation of the mtDNA D-loop region and downregulation of oxidative phosphorylation (OXPHOS) complexes, consequently, leading to metabolic reprogramming in NPC. Furthermore, we demonstrate that grifolin, a natural farnesyl phenolic compound originated from higher fungi, is able to attenuate glycolytic flux and recover mitochondrial OXPHOS function by inhibiting DNMT1 expression and activity as well as its mitochondrial retention in NPC cells. Therefore, our work establishes a mechanistic connection between epigenetics and metabolism in EBV-positive NPC and provides further evidence for pathological classification based on CpG island methylator phenotype (CIMP) in EBV-associated malignancies. In addition, grifolin might be a promising lead compound in the intervention of high-CIMP tumor types. The availability of this natural product could hamper tumor cell metabolic reprogramming by targeting DNMT1.

## Introduction

Epstein–Barr virus (EBV) is the first human oncogenic virus that contributes to a wide variety of malignancies of both lymphoid and epithelial origin, such as Burkitt’s and Hodgkin’s lymphomas, nasopharyngeal carcinoma (NPC) and gastric carcinoma (GC)^1^. EBV infection is characterized by the expression of latent genes including Epstein–Barr nuclear antigens (EBNAs and EBNA leader protein (EBNA-LP)), latent membrane proteins LMP1 and LMP2 and the non-coding EBV-encoded RNAs (EBERs) and viral microRNA (miRNA)^1^. Among them, LMP1 is defined as a driver oncogene in NPC and plays an important role in NPC pathogenesis due to triggering multiple cell signaling pathways, which promote cell transformation, proliferation, immune escape, invasiveness, epigenetic modification, and metabolic reprogramming^2–10^.

CIMP is defined as the high activity of global and nonrandom CpG island methylation. EBV infection is an epigenetic driver and closely associated with CIMP^1,3,11^. CpG island promoter methylation of tumor suppressor genes is one of the most characteristic abnormalities in EBV-associated malignancies^12–14^. The unbiased genome-scale analysis of NPC methylome displayed a high-degree CpG methylation epigenotype. Epigenetic disruption of Wnt, MAPK, TGF-β and Hedgehog signaling pathways, among others, were identified using methylated DNA immunoprecipitation coupled with promoter microarray hybridization (MeDIP-chip)^14^. Similarly, EBV-associatedMS was remarkable among the cases of CIMP-high GC^15–17^. The DNA methyltransferases (DNMTs) link CpG methylation, chromatin remodeling, and subsequent gene silencing, and DNMT1 is responsible for methylating hemimethylated DNA and DNA methylation maintenance thereof^18,19^. Phosphatase and tensin homolog (PTEN) is a tumor suppressor and functions as an important negative regulator of aerobic glycolytic programs^20–22^. Epigenetic silencing of the *pten* gene contributes to PTEN inactivation in multiple types of cancers^23–27^. The methylation level of *pten* in NPC specimens reached 82.2% relative to 5.3% in nasopharyngeal tissues^27^. InMS cells infected with recombinant EBV, activation of DNMT1 by LMP2A was reported to enhance promoter hypermethylation of the *pten* gene^12^.

Cancer cells adapt their metabolism to support tumorigenicity and malignancy^28,29^. Cellular transformation is characterized by reduced OXPHOS and increased aerobic glycolysis, and cells rapidly increase glucose utilization and lactate production regardless of oxygen availability^29^. Aerobic glycolysis facilitates rapid cell division by providing both energy and metabolic intermediates for the anabolic biosynthesis of macromolecules^29^. Epigenetics and metabolism are closely interconnected in a reciprocal fashion^30^. The expression and activity of various metabolic enzymes are found to be altered not only by genetic mutations but also epigenetic mechanisms such as hypo or hypermethylation of the promoter region and acetylated modification^31–33^. The mitochondrial OXPHOS system consists of both nuclear- and mitochondrial DNA (mtDNA)-encoded subunits^34^. Mitochondrial genome contains a non-coding region including a unique displacement loop (D-loop), which is responsible for replication and transcription of mtDNA^35^. The presence of methylated cytosine residues within mtDNA have been reported. Shock et al.^36^ observed the translocation of DNMT1 into the mitochondria and confirmed an enrichment of mtDNA sequences by immunoprecipitation against 5-methylcytosine (5mC)^36^.

Several natural compounds such as polyphenols, flavonoids, antraquinones exhibit potent inhibitory effects on DNMT activity and/or expression, thus displaying demethylation and re-activation of genes associated with tumor progression^37,38^. Grifolin, a farnesyl phenolic compound, is a secondary metabolite derived from the mushrooms *Albatrellus confluens* and *Boletus pseudocalopus*^39–41^. Grifolin exhibits various microbiological and pharmacological effects^42,43^. The anticancer activities of grifolin were first reported by our group and the previous studies showed that grifolin inhibited the growth, invasion, and metastasis of multiple types of tumor cells^44–50^.

In the present study, we evaluated the role of DNMT1 in mediating metabolic reprogramming of NPC induced by LMP1. Based on the results, the underlying mechanism was further investigated. Moreover, the effect of grifolin on glycolytic flux and mitochondrial OXPHOS by targeting DNMT1 was explored. The aim of the present study is to clarify the mechanistic connection between epigenetics and metabolism in EBV-positive NPC and further develop novel pharmacological tools in the intervention of tumors with high CIMP by targeting DNMT1.

## Results

### LMP1 regulates glycolysis in nasopharyngeal carcinoma

**Fig. 1:**
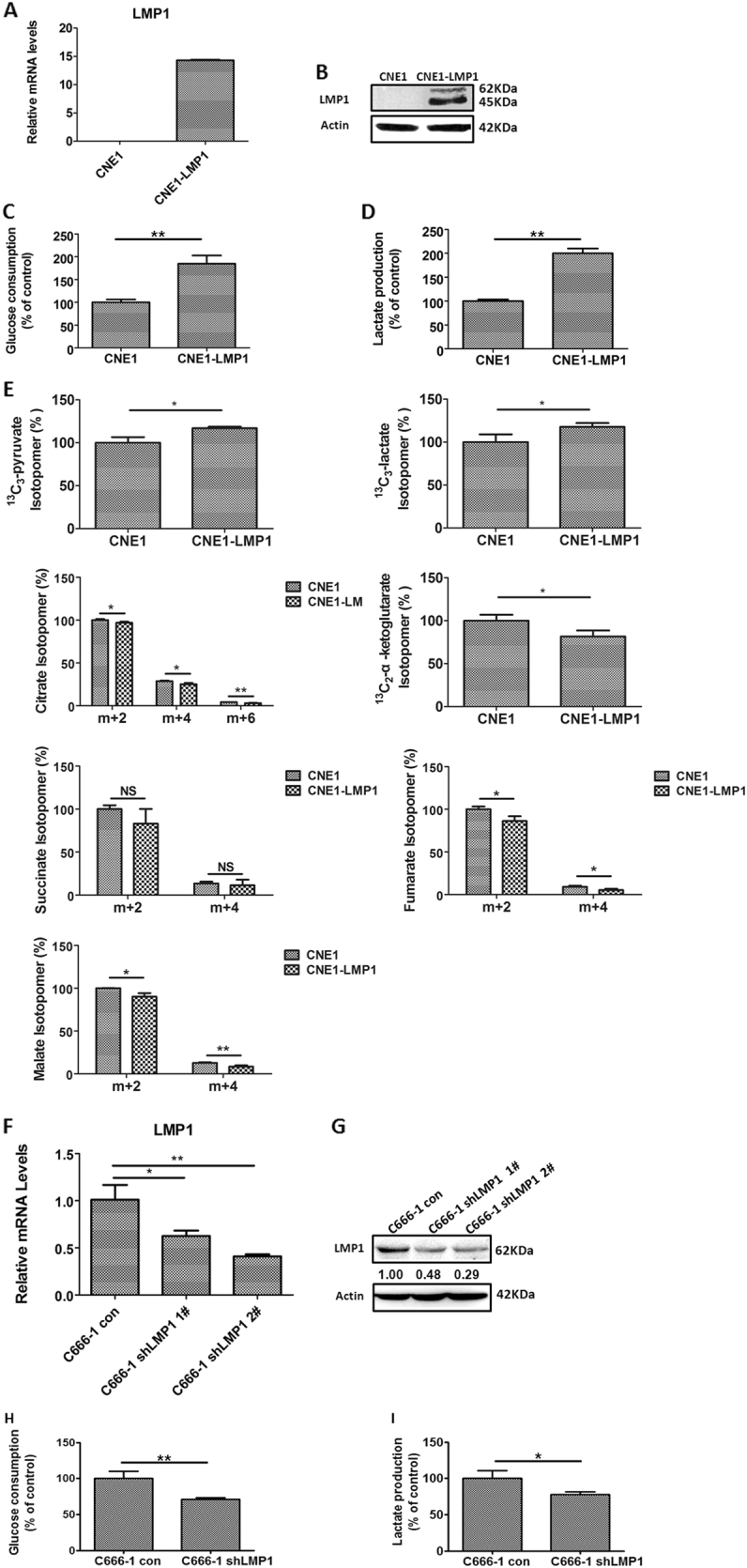
LMP1 promotes reprogramming of glucose metabolism in NPC cells. **a** The mRNA and (**b**) protein levels of LMP1 in CNE1 and CNE1-LMP1 cells. **c** Glucose consumption and (**d**) lactate generation in CNE1 and CNE1-LMP1 cells. **e** GC/MS-based metabolome analysis of the indicated metabolite levels after labeling CNE1 and CNE1-LMP1 cells with ^13^C_6_-d-glucose (“*m*” represents the mass of the metabolite fragment ion without any ^13^C; “*m* + *n*” (*n* = 2, 4, 6), where “*n*” represents the number of ^13^C atoms in the metabolite). **f** The mRNA and (**g**) protein levels of LMP1 in C666-1 control (con) or C666-1 shLMP1 cells. **h** Glucose consumption and (**i**) lactate generation in C666-1 con or C666-1 shLMP1 cells. Data are shown as mean values ± S.D. of independent, triplicate experiments. The asterisks (*, **) indicate significant differences (*p* < 0.05, *p* < 0.01, respectively). NS not significant (Student’s *t* test)

To gain insight into the role of LMP1 in metabolic reprogramming of NPC, we determined both glucose consumption and lactate production in LMP1-negative CNE1 cells and LMP1-overexpressing CNE1-LMP1 cells, respectively. We found that glycolytic flux in CNE1-LMP1 cells was markedly enhanced by about 90% compared to that in CNE1 cells (Fig. 1a–d). Furthermore, metabolic flux measurements were performed. CNE1 and CNE1-LMP1 cells were treated with ^13^C_6_-d-glucose for 2 h and then extracted metabolites were applied for metabolome analysis by Gas chromatography/Mass spectrometer (GC/MS). We found that ^13^C-labeled pyruvate and lactate levels were markedly elevated, whereas ^13^C-labeled metabolite levels of the tricarboxylic acid cycle, such as ^13^C-citrate, ^13^C-α-ketoglutarate, ^13^C-fumarate, or ^13^C-malate, were significantly decreased in CNE1-LMP1 cells compared to CNE1 cells (Fig. 1e). NPC C666-1 cells consistently harbor EBV^51^. We established stable C666-1 shLMP1 1# and 2# cell lines and confirmed that the shLMP1 2# exhibited higher knockdown efficiency of LMP1 (>70%; Fig. 1f, g). Accordingly, we employed the shLMP1 2# cell line for further investigations. We observed that genetic depletion of LMP1 in EBV-positive C666-1 cells led to decreases in glycolysis (Fig. 1h, i). These findings clearly demonstrated that LMP1 promotes glucose metabolism from oxidative phosphorylation to aerobic glycolysis in NPC cells.

### DNMT1 mediates the downregulation of PTEN by LMP1 to activate AKT signaling

**Fig. 2:**
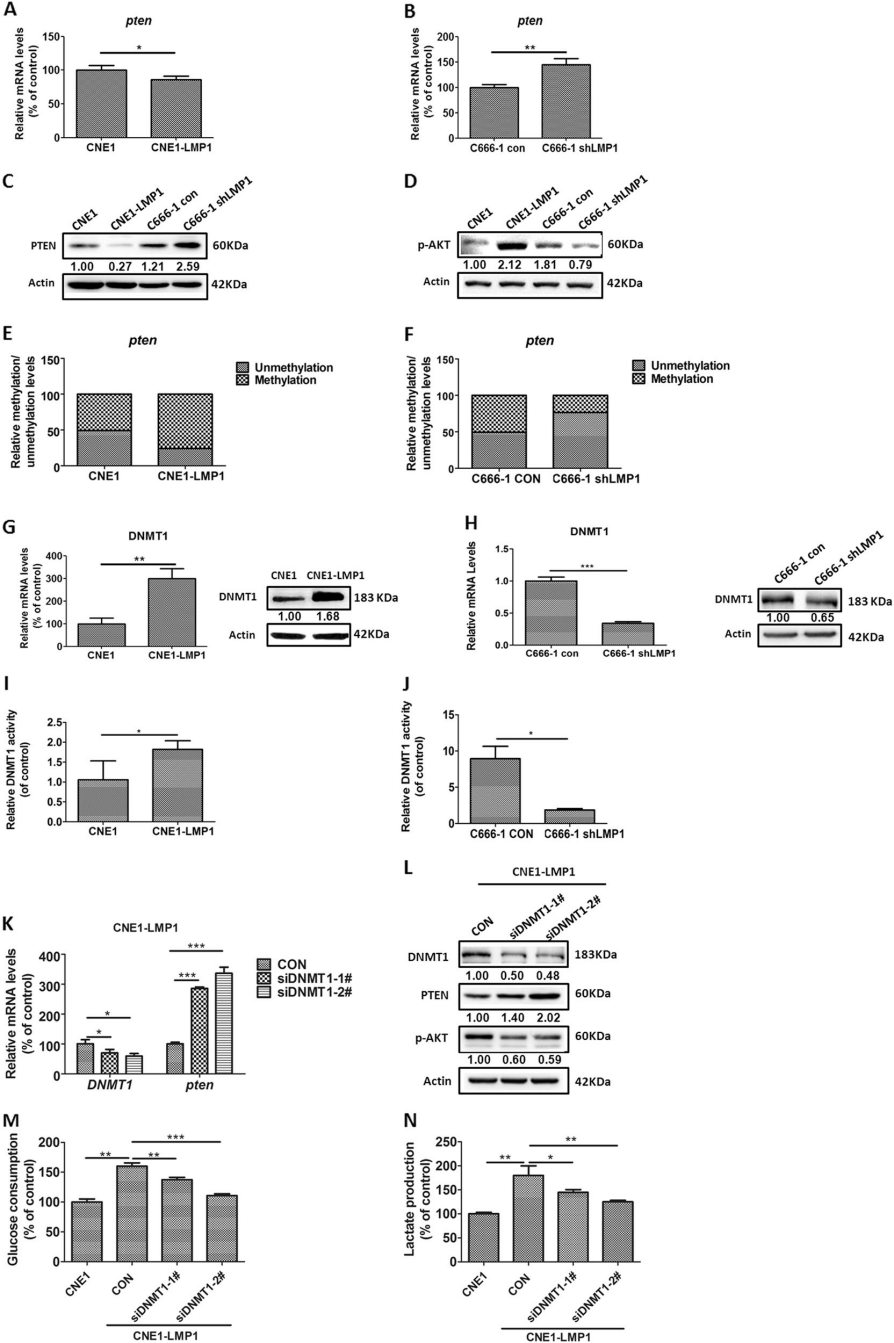
DNMT1 mediates the downregulation of PTEN by LMP1. The mRNA levels of the *pten* gene in (**a**) CNE1 and CNE1-LMP1 cells and (**b**) C666-1 con and C666-1 shLMP1 cells. The protein levels of (**c**) PTEN and (**d**) p-AKT in CNE1 and CNE1-LMP1 cells and C666-1 con and C666-1 shLMP1 cells. Methylated and unmethylated levels of the *pten* gene in (**e**) CNE1 and CNE1-LMP1 cells and (**f** ) C666-1 con and C666-1 shLMP1 cells. The mRNA and protein levels of DNMT1 in (**g**) CNE1 and CNE1-LMP1 cells and (**h**) C666-1 con and C666-1 shLMP1 cells. DNMT1 activity levels in (**i**) CNE1 and CNE1-LMP1 cells and ( **j**) C666-1 con and C666-1 shLMP1 cells. **k** The mRNA levels of *pten* and *DNMT1* genes in CNE1-LMP1 cells treated with control siRNA (CON) or 2 different DNMT1 siRNAs (1# and 2#). **l** The protein levels of DNMT1, PTEN, and p-AKT were detected by western blot assay in CNE1 and CNE1-LMP1 cells. **m** Glucose consumption and (**n**) lactate generation in CNE1 and CNE1-LMP1 cells treated with control siRNA or DNMT1 siRNAs (1# and 2#). Data are shown as mean values ± S.D. of independent, triplicate experiments. The asterisks (*, **, ***) indicate significant differences (*p* < 0.05, *p* < 0.01, *p* < 0.001, respectively)

The loss of function of *pten* leads to oncogenetic AKT signaling activation, which plays important roles in carbohydrate metabolism^22,52^. In order to further clarify the underlying mechanism in LMP1-induced avidity of aerobic glycolysis, we examined the function of LMP1 on PTEN expression and the downstream phosphorylation levels of AKT. In LMP1-overexpressing NPC cells, PTEN was substantially suppressed both at the mRNA and protein levels (Fig. 2a–c), and the downstream p-AKT levels were markedly increased compared to the control (Fig. 2d). In contrast, genetic depletion of LMP1 enhanced PTEN expression (Fig. 2b, c) and decreased p-AKT levels (Fig. 2d).

Next, a methylation-specific PCR (MSP) assay was performed to investigate the effect of LMP1 on genomic methylation near the *pten* transcription start site. We observed that LMP1 overexpression augmented the methylation/unmethylation (M/U) ratio of genetic *pten* DNA (Fig. 2e). In contrast, knocking down LMP1 reversed the ratio compared to the control (Fig. 2f). Also, the methylation status of *pten* DNA inversely corresponded to mRNA and protein expression in NPC cells. To confirm the function of methyltransferases in PTEN silencing, we examined the influence of LMP1 on the maintenance methyltransferase, DNMT1. Results showed that LMP1 promoted both DNMT1 expression and enzymatic activity (Fig. 2g–j). Furthermore, knocking down DNMT1, using 2 different siRNAs (siDNMT1-1# and siDNMT1-2#), in LMP1-expressing NPC cells rescued PTEN expression and suppression of AKT pathway (Fig. 2k, l, Supplementary Figure 1), and hampered aerobic glycolysis to a degree similar to that observed in LMP1-negative CNE1 cells (Fig. 2m, n).

Overall, these results support the idea that LMP1 downregulates PTEN/AKT signaling to activate glycolytic flux in a methylation-dependent manner, and that DNMT1 plays an essential role in mediating these processes.

### LMP1 modulates DNMT1 mitochondrial localization to inhibit OXPHOS

**Fig. 3:**
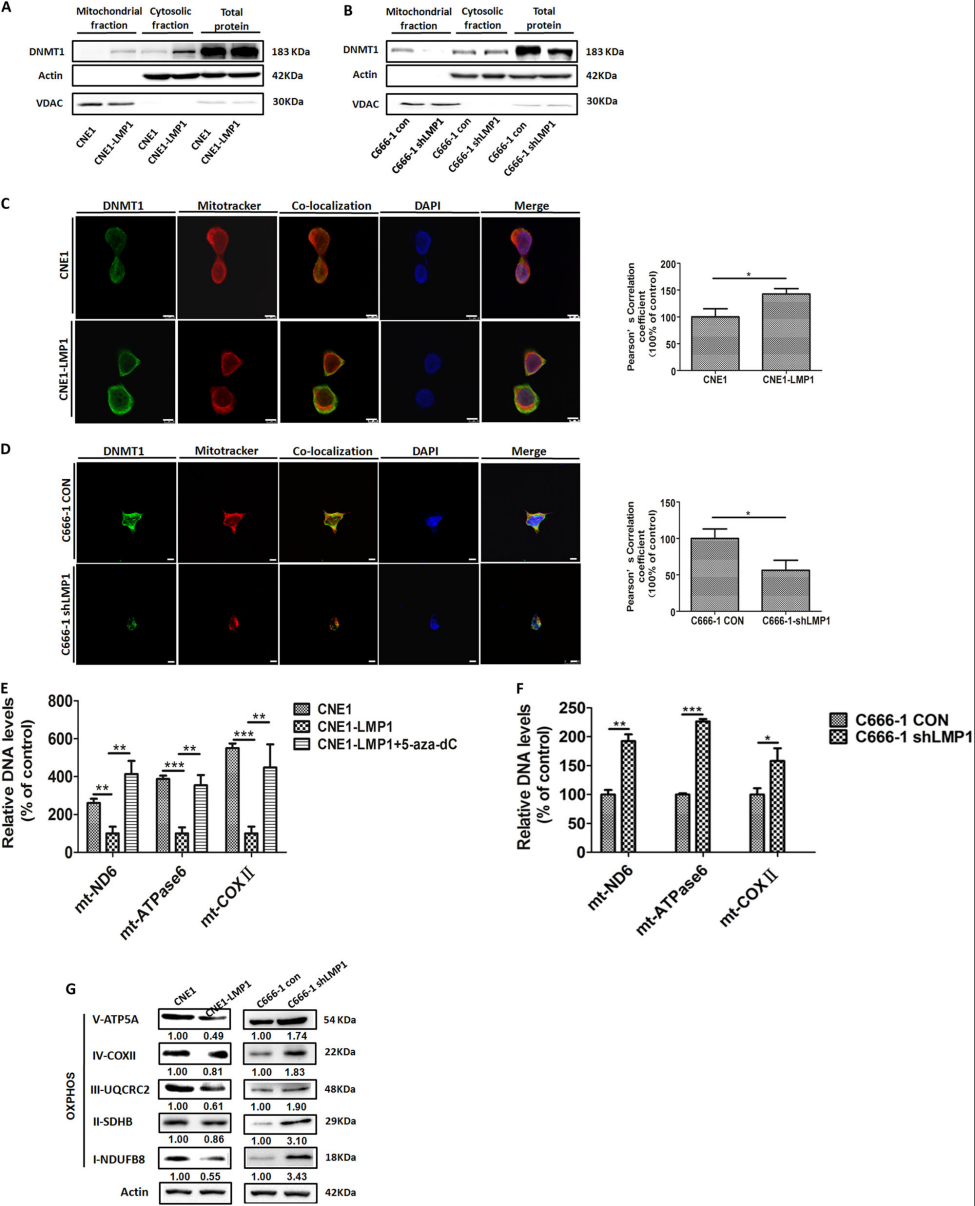
LMP1 promotes DNMT1 mitochondrial localization to inhibit OXPHOS complex genes expression. The expression levels of DNMT1 in mitochondrial fractions, cytosolic fractions, and total cell lysates of (**a**) CNE1 and CNE1-LMP1 cells and (**b**) C666-1 con and C666-1 shLMP1 cells. VDAC serves as a mitochondria-specific marker and actin is a loading control for western blot analysis. **c** CNE1 and CNE1-LMP1 cells and (**d**) C666-1 con and C666-1 shLMP1 cells were seeded on coverslips overnight and the co-localization of DNMT1 (green) and mitochondria (red) was determined by confocal microscopy, when nuclei were stained blue. Mitochondria were stained using Mitotracker. Pearson’s correlation coefficients for the co-localization of DNMT1 and mitochondria are shown as bar graphs (scale bar, 10 μm). **e** The DNA levels of mitochondria-encoded genes mt-ND6, mt-ATPase6, and mt-COXII in each designated group. In the CNE1-LMP1 + 5-aza-dC group, cells were incubated in basal medium containing 5% serum containing 5-aza-dC (10 μM) for 5 days. **f** The DNA levels of mt-ND6, mt-ATPase6, and mt-COXII in C666-1 con and C666-1 shLMP1 cells. **g** The protein levels of OXPHOS complex proteins in CNE1 and CNE1-LMP1 and C666-1 con and C666-1 shLMP1 cells. Data are shown as mean values ± S.D. of independent, triplicate experiments. The asterisks (*, **, ***) indicate significant differences (*p* < 0.05, *p* < 0.01, *p* < 0.001, respectively)

DNMTs can enter into mitochondria to regulate mtDNA transcription^36^. To investigate whether LMP1 modulates DNMT1 mitochondrial translocation, we examined DNMT1 expression in mitochondrial fractions, cytosolic fractions, and total protein extracts, respectively. Notably, we found that in LMP1-overexpressing NPC cells, mitochondrial DNMT1 was markedly augmented compared to LMP1-negative cells (Fig. 3a). Conversely, knocking down LMP1 attenuated DNMT1 retention in mitochondria (Fig. 3b). Moreover, we used an immunofluorescence assay to determine the organelle localization of DNMT1 in differentiated LMP1-expressing NPC cells. Mitotracker served as a mitochondrial probe (Fig. 3c, d). Computerized quantification of fluorescent co-localization between mitochondria and DNMT1 was completed. These findings indicated that LMP1 promoted DNMT1 mitochondrial localization, which was consistent with the observations in mitochondrial protein fractions (Fig. 3a, b). In addition, we demonstrated that the translocation of DNMT1 driven by LMP1 is exclusive to mitochondria and not other fractions, such as endoplasmic reticulum (Supplementary Figure 2).

Given that DNMTs participate in the establishment of the mtDNA control region (D-loop) methylation pattern^36^, they might affect mitochondria-encoded gene expression, such as the OXPHOS complex genes. First, the MSP assay revealed that LMP1 markedly increased the M/U ratio of DNA fragments spanning the mitochondrial D-loop region (Fig. 6a, lane 1 vs. 4). Next, we found that LMP1 overexpression hampered mt-ND6 (ubiquinone oxidoreductase core subunit 6), mt-ATPase6 (ATP synthase membrane subunit 6), and mt-COXII (cytochrome c oxidase subunit II) DNA levels (Fig. 3e). In contrast, genetic depletion of LMP1 restored their expression (Fig. 3f). Moreover, treatment with the hypomethylating agent, 5-aza-2′-deoxycytidine (5-aza-dC), a well-known inhibitor of DNMT1, substantially reduced the ability of LMP1 to restrain OXPHOS complex gene transcription (Fig. 3e). The inhibition of OXPHOS complex protein expression induced by LMP1 further confirmed this finding (Fig. 3g). Altogether, these experiments support the notion that (1) LMP1 promotes DNMT1 mitochondrial translocation and (2) LMP1 regulates OXPHOS complex gene expression in a DNMT1-dependent manner.

### Grifolin targets DNMT1 to inhibit aerobic glycolysis

**Fig. 4:**
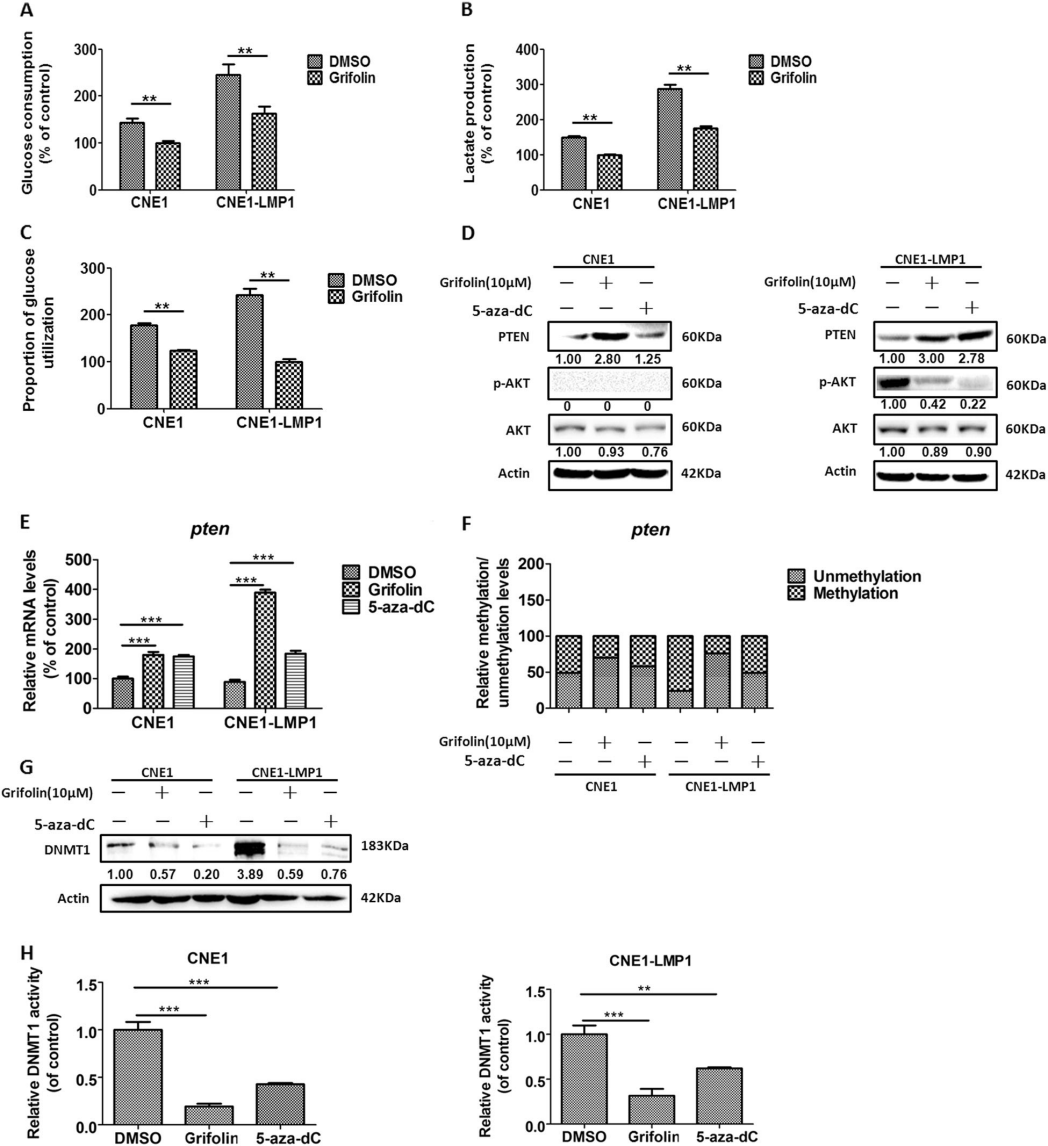
Grifolin targets DNMT1 to inhibit glycolysis. Grifolin treatment inhibits (**a**) glucose consumption and (**b**) lactate generation, and (**c**) the proportion of glucose utilization in CNE1 and CNE1-LMP1 cells. **d** Effect of grifolin on PTEN expression and phosphorylation level of AKT in CNE1 and CNE1-LMP1 cells. Effect of grifolin on (**e**) mRNA and (**f** ) methylated/unmethylated levels of the *pten* gene in CNE1 and CNE1-LMP1 cells. Grifolin treatment decreases DNMT1 (**g**) protein expression and (**h**) enzymatic activity in CNE1 and CNE1-LMP1 cells. CNE1 and CNE1-LMP1 cells were incubated in basal medium containing 5% serum with DMSO, grifolin (10 μM), or 5-aza-dC (10 μM) for 5 days. Data are shown as mean values ± S.D. of independent, triplicate experiments. The asterisks (**, ***) indicate significant differences (*p* < 0.01, *p* < 0.001, respectively)

Epigenetic agents, including DNMT inhibitors (DNMTIs) and histone deacetylase (HDAC) inhibitors, have provided tremendous promise for the treatment of advanced, metastatic cancer by episensitization to overcome chemotherapy resistance^53,54^. Natural compounds such as polyphenols, flavonoids, and antraquinones represent an important source for epidrug candidates^38^. In order to determine whether phenolic grifolin acts as a DNMT1 inhibitor to block LMP1-augmented glycolysis, we first examined glycolytic flux with grifolin treatment. In CNE1 and CNE1-LMP1 cells, both glucose consumption and lactate production were significantly inhibited with grifolin treatment compared to the DMSO control (Fig. 4a, b). In the process of glycolysis, one mole of glucose generates two moles of lactate (Fig. 4b). We converted the proportion of glucose used for lactate production to total glucose consumption and found that grifolin treatment markedly decreased the ratio compared to the DMSO control. Moreover, in CNE1-LMP1 cells, the ratio of grifolin-treated group is much lower than that of CNE1 cells (Fig. 4c). These findings indicate that grifolin treatment hampers glycolytic flux more effectively in LMP1-positive cells.

We next examined PTEN and p-AKT expression in each designated group. 5-aza-dC served as a positive control. As expected, grifolin treatment significantly reduced p-AKT levels (Fig. 4d) and enhanced PTEN mRNA as well as protein levels both in CNE1 and CNE1-LMP1 cells (Fig. 4d, e). The effect was more marked in CNE1-LMP1 cells, in which DNMT1 was highly augmented by LMP1 (Fig. 2h–k). MSP analysis revealed that treatment with grifolin reduced the methylation/unmethylation (M/U) ratio of *pten* DNA especially in LMP1-positive cells (Fig. 4f), which was consistent with the changes in mRNA and protein levels. Furthermore, we found that treatment of grifolin markedly attenuated both protein expression and enzymatic activity of DNMT1 (Fig. 4g, h). Moreover, the effects of grifolin were much similar to those of 5-aza-dC treatment. Therefore, these findings show that grifolin inhibits glycolysis by targeting DNMT1 to demethylate and reactivate the *pten* gene in NPC cells.

### Grifolin blocks DNMT1 mitochondrial localization to restore OXPHOS

**Fig. 5:**
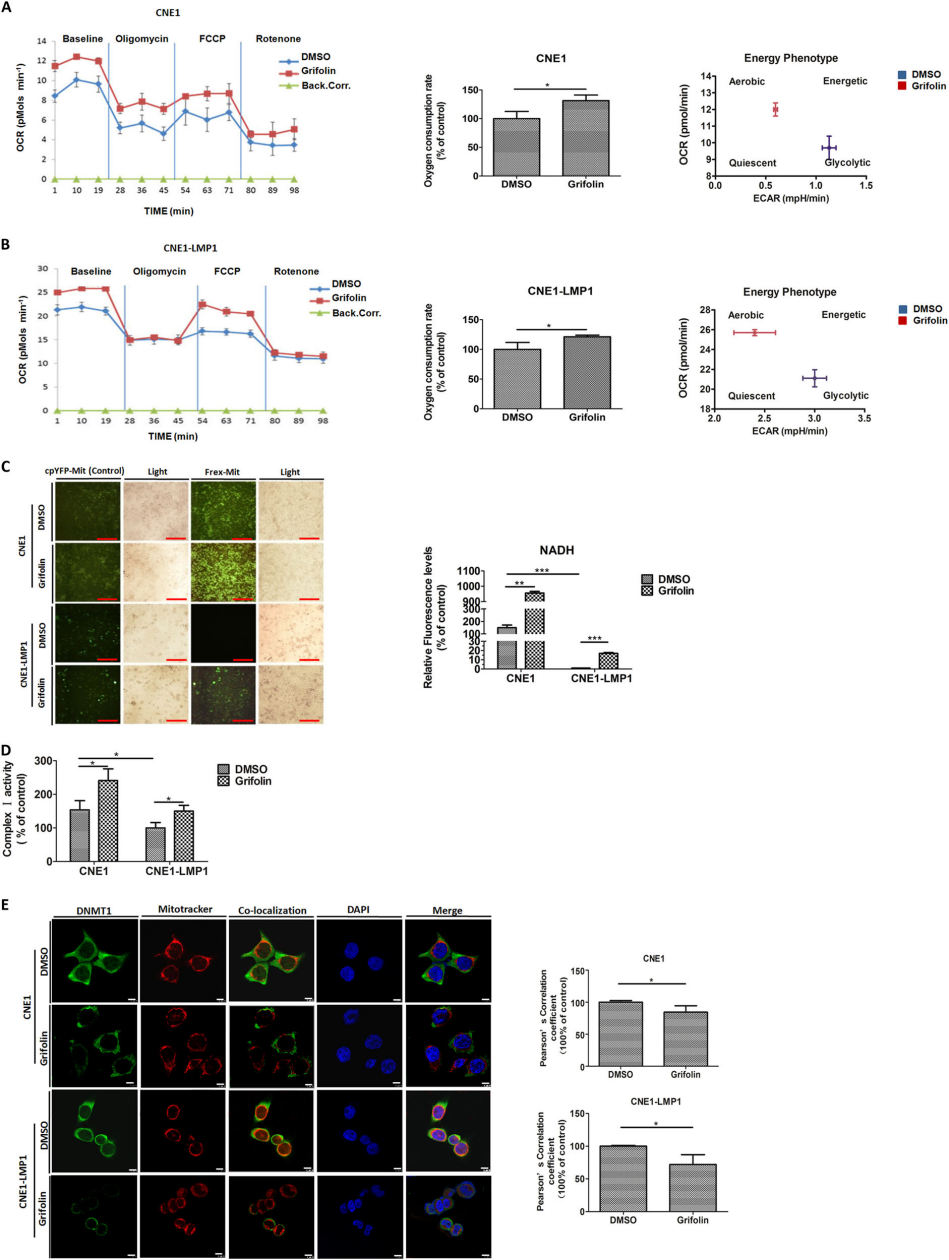
Grifolin blocks DNMT1 mitochondrial localization to restore OXPHOS. Mitochondrial respiration was determined by measuring oxygen consumption rate (OCR) without (DMSO) or with grifolin treatment in (**a**) CNE1 and (**b**) CNE1-LMP1 cells. Left, seahorse extracellular flux analyzer measurements of OCR metabolic profile using the mito stress cell assay. Traces shown are representative of two independent experiments in which each data point represents replicates of five wells. Data are shown as mean values ± S.D. Middle, quantitative determination of basal OCR value of each designated group. Right, cellular energy phenotype of each designated group. **c** CNE1 and CNE1-LMP1 cells were treated with DMSO or grifolin (10 μM) for 3 days and intracellular NADH levels were detected using NADH-specific sensor Frex-Mit at the excitation wavelength of 485 nm. cpYFP-Mit was used as a negative control and the light imaging of each field as cell density control. Quantification of the fluorescent intensity of Frex-Mit imaging is shown as bar graphs. **d** Grifolin treatment rescues CI activity downregulated by LMP1. CNE1 and CNE1-LMP1 cells were treated with DMSO or grifolin (10 μM) for 5 days and CI activity was measured. **e** Grifolin treatment inhibits translocation of DNMT1 to mitochondria. CNE1 and CNE1-LMP1 cells were seeded on coverslips overnight and treated with DMSO or grifolin (10 μM) for 5 days, respectively. The co-localization of DNMT1 (green) and mitochondria (red) was detected by confocal microscopy, and nuclei are stained blue. Pearson’s correlation coefficients for the co-localization of DNMT1 and mitochondria are shown as bar graphs. Scale bar, 10 μm. Data are shown as mean values ± S.D. of independent, triplicate experiments. The asterisks (*, **, ***) indicate significant differences (*p* < 0.05, *p* < 0.01, *p* < 0.001, respectively)

Because we have previously shown that LMP1 modulates DNMT1 mitochondrial localization to inhibit OXPHOS, we further hypothesized that, except for its inhibitory effect on glycolysis, grifolin might restore OXPHOS by targeting DNMT1. To test this hypothesis, we examined the change in oxygen consumption rate in the absence or presence of grifolin in CNE1 and CNE1-LMP1 cells, respectively. The results showed that, compared to the DMSO control, grifolin treatment enhanced mitochondrial basal oxygen respiration capacities both in CNE1 and CNE1-LMP1 cells, and the plotted metabolic phenograms further verified the switch from glycolytic to aerobic metabolism after grifolin treatment (Fig. 5a, b). In mitochondria, tricarboxylic acid (TCA) cycle reactions drive the formation of NADH and FADH_2_, which then transfer their electrons to the electron transport chain (ETC) to generate ATP. Because NADH represents an important intermediate metabolite of mitochondrial OXPHOS, we next determined the effect of grifolin on cellular NADH levels. Frex-Mit is a highly sensitive NADH sensor of living cells. Cells transfected with the cpYFP-Mit plasmid are set as a negative control, and the light-field images represent the same cell density in each group. We observed that the fluorescence of cells transfected with the Frex-mit plasmid was dramatically increased by treatment with grifolin compared to the DMSO group, especially in LMP1-positive cells. Computerized quantification of average fluorescent intensity of images in each group was performed (Fig. 5c). Complex I (CI) is the major entry point of electrons into the respiratory chain that oxidizes NADH to provide the ETC with two electrons per molecule of NADH. We further determined CI activities in cells with or without grifolin treatment. The results demonstrated that compared to CNE1, CI activities were markedly reduced in CNE1-LMP1 cells and grifolin treatment resulted in over 50% increase in the activities of both CNE1 and CNE1-LMP1 cells (Fig. 5d). LC-MS/MS analysis further showed that the grifolin-treated group recruited more species of OXPHOS complex subunits in comparison to the DMSO control in both CNE1 and CNE1-LMP1 cells (Supplementary Tables 1, 2). These experiments indicate that LMP1 downregulates CI activity and this can be rescued by grifolin treatment, which promotes subunit assembly to form OXPHOS complexes and ensures high level of NADH actually used by CI for augmentation of mitochondrial OXPHOS activity.

To further address the proposed hypothesis, an immunofluorescence assay was performed to verify the effect of grifolin on DNMT1 mitochondrial localization. We observed that treatment with grifolin significantly attenuated the retention of DNMT1 in mitochondria compared with the DMSO control in both CNE1 and CNE1-LMP1 cells. Computerized quantification of fluorescent co-localization between mitochondria and DNMT1 confirmed this finding (Fig. 5e).

**Fig. 6:**
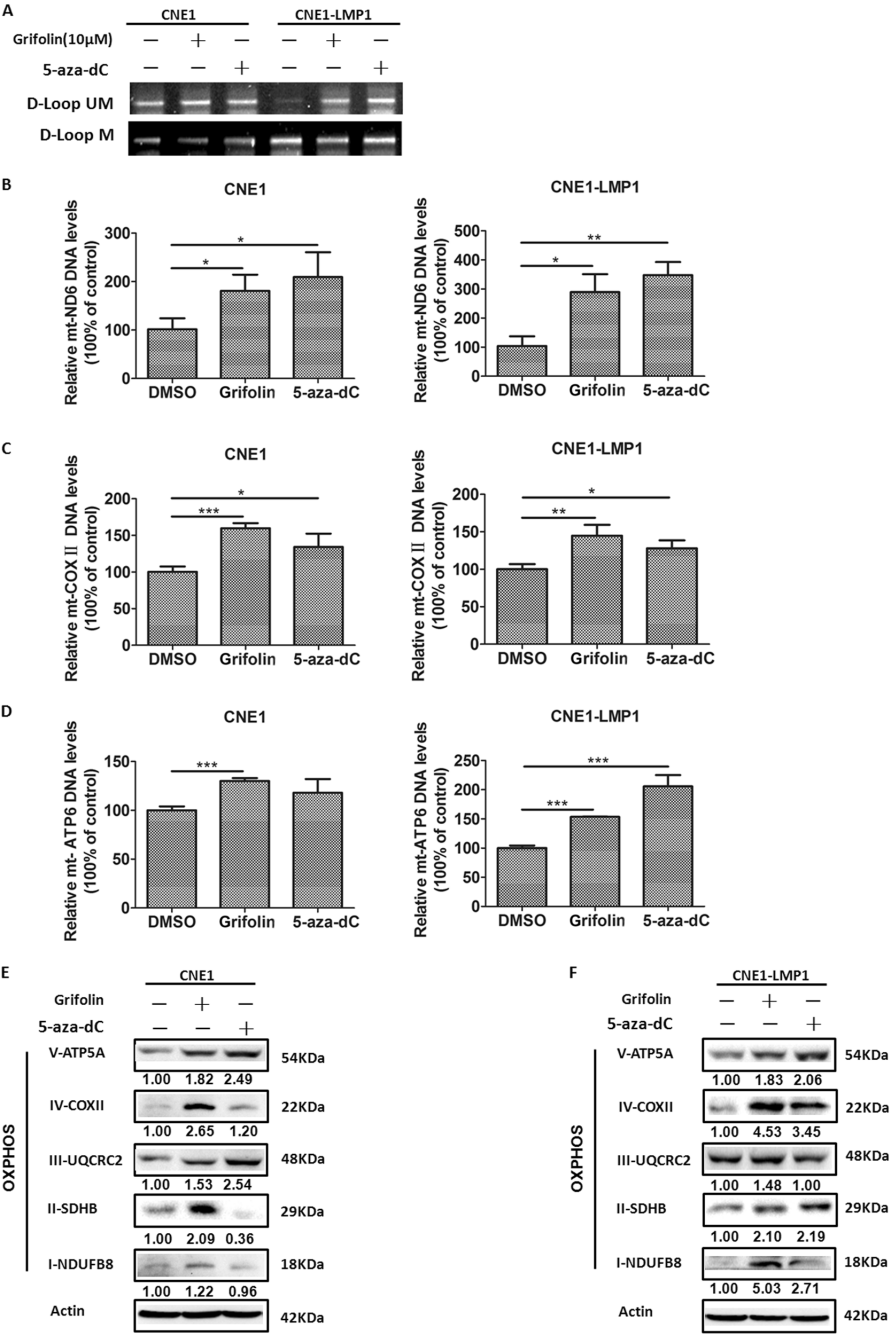
Grifolin treatment restores OXPHOS complexes expression. CNE1 and CNE1-LMP1 cells were treated with DMSO, 5-aza-dC (10 μM) or grifolin (10 μM) for 5 days. **a** The methylated/unmethylated levels of the D-loop region in mitochondrial DNA was determined by MSP analysis. **b**–**d** The DNA levels of mitochondria-encoded *ND6*, *COXII*, and *ATPase6* genes were examined by real-time PCR in each designated group in CNE1 and CNE1-LMP1 cells. Data are shown as mean values ± S.D. of independent, triplicate experiments. The asterisks (*, **, ***) indicate significant differences (*p* < 0.05, *p* < 0.01, *p* < 0.001, respectively). The OXPHOS complex I–V proteins were detected by western blot analysis in each designated group in (**e**) CNE1 and (**f**) CNE1-LMP1 cells. UM unmethylation, M methylation

Notably, we found that similar to the 5-aza-dC group, the M/U ratio of the mtDNA D-loop region was markedly reduced in the grifolin-treated group (Fig. 6a). To further evaluate the demethylation effect of grifolin on mitochondria-encoded genes, we examined the DNA levels of representative OXPHOS complex genes in each designated group. The experiments showed that grifolin treatment effectively enhanced the genes levels of mitochondrial ATP6, COXII, and ND6 (Fig. 6b–d). Similar results were obtained for the 5-aza-dC group. Moreover, the protein expression of OXPHOS complexes confirmed this observation (Fig. 6e, f, Supplementary Figure 3A, 3B). In addition, treatment with grifolin or 5-aza-dC did not affect the levels of LMP1 in CNE1-LMP1 cells (Supplementary Figure 3C), which excluded the possibility that the effect of grifolin to restore OXPHOS complex gene expression restrained by LMP1 might be due to the downregulation of LMP1 expressions. Moreover, no modifications in mitochondrial morphology were observed after grifolin treatment in both CNE1 and CNE1-LMP1 cells, which indicated that grifolin had no effect on mitochondrial dynamics (Supplementary Figure 4). Collectively, these findings indicate that treatment with grifolin targets DNMT1 and causes demethylation of the mtDNA D-loop region and reactivates mitochondria-encoded OXPHOS complex genes to enhance mitochondrial respiration capacity.

## Discussion

Cancer cells frequently display fundamentally altered cellular metabolism, which provides biochemical foundation and directly contributes to malignant progression^55–57^. Cellular epigenetic and metabolic networks are highly interactive. On one hand, activities of many chromatin-modifying enzymes can be modulated by the concentrations of their specific metabolic substrates or co-factors, including ten–eleven translocation (TET) proteins^58^, histone acetyltransferases (HATs), and HDAC^59^. On the other hand, epigenetic alterations of metabolic-related enzymes leads to altered metabolic flux that is advantageous to cancer cells. Hypomethylation of the hexokinase isoform 2 (*HK2*) gene promoter is observed in liver cancer and glioblastoma, which results in upregulation of HK2 and elevated glycolytic flux^31^. While acetylation of pyruvate kinase M2 (PKM2) decreases its activity and facilitates glycolytic flux into macromolecules biosynthesis^32^.

Oncoviral infections drive distinctive patterns of epigenetic modification in cancer. Viral oncoproteins such as adenovirus small E1A (e1a), human papillomavirus (HPV) oncoproteins E2, E6, and E7 can employ epigenetic modifiers to mediate host cell transformation and malignancy^60–63^. Some studies have shown that EBV-encoded products, including LMPs, BZLF1, EBNA1-2, BRLF1, interact with epigenetic modifying enzymes, such as DNMT1, p300/CBP, PCAF, p400, and GCN5, to facilitate cell development and maintenance of EBV-associated cancer^3,11,12,64,65^. As a potent oncogenic protein encoded by EBV, LMP1 builds a natural bridge between epigenetic modification and metabolic change that are closely associated with the EBV-driven pathogenesis of NPC. In a previous study from our group, we demonstrated that LMP1-induced upregulation of HK2 promoted glycolysis and enhanced resistance of NPC to radiotherapy^7^. Lo et al.^8^ illustrated that LMP1 promotes aerobic glycolysis and transformation of human nasopharyngeal epithelial cells through activation of the FGF2/FGFR1 signaling pathway. Zhang et al.^66^ found that LMP1 upregulated glucose transporter-1 (GLUT1) through the mTORC1/NF-κB signaling pathways. In the present study, from the perspective of epigenetic regulation, we reported for the first time that DNMT1 mediates metabolic reprogramming induced by LMP1. Our findings first reveal that LMP1 not only upregulates DNMT1 expression and activity, but also promotes its mitochondrial localization, which leads to hypermethylation of the mtDNA D-loop region and mtDNA-encoded OXPHOS complex genes downregulation. Thereby, DNMT1 mediates both the enhancement of glycolysis and damage to mitochondrial respiration induced by LMP1.

Epigenetic changes usually occur in the early stage of tumorigenesis and appear reversible, which provides the possibility for chemoprevention^67,68^. The nucleoside analogs, vidaza and dacogen (5-aza-dC), are currently the only epidrugs approved by the FDA. Nevertheless, they are chemically unstable and have serious side effects^38^. Thereby, identifying new nucleoside-based or novel structure-based candidates targeting DNMTs is challenging. Polyphenols derived from natural compounds are among the most effective species for DNMT inhibitors^69^. Here, we demonstrate that phenolic grifolin is able to attenuate glycolytic flux and recover OXPHOS by inhibiting DNMT1 expression and activity as well as its mitochondrial retention in NPC cells. Grifolin treatment causes demethylation of the *pten* gene and blocks activation of AKT signaling, which leads to inhibition of aerobic glycolysis. Moreover, the attenuation of DNMT1 retention in mitochondria by grifolin effectively causes demethylation of the mtDNA D-loop region and, consequently, restores mitochondria- encoded OXPHOS gene expressions and enhanced oxygen respiration.

In conclusion, given that CIMP has been used in the classification of EBV-positive tumors, our work provides new evidence for pathological classification based on CIMP in EBV-associated malignancies and establishes a mechanistic connection between epigenetics and metabolism in EBV-positive NPCs. In addition, grifolin might represent a promising candidate lead compound in the intervention of high-CIMP tumor types. The availability of this natural product will hamper tumor cell metabolic reprogramming by targeting DNMT1.

## Materials and methods

### Cell culture

The human NPC CNE1, CNE1-LMP1, and C666-1 cells have been described previously^7,51^. All cells were grown in RPMI 1640 media supplemented with 10% v/v heat-inactivated fetal bovine serum, 1% w/v glutamine, 1% w/v penicillin and streptomycin, and cultured at 37 °C in a humidified incubator containing 5% CO_2_.

### Reagents and chemicals

The antibodies against β-actin and LMP1 were purchased from Sigma-Aldrich (St. Louis, MO, USA) and DAKO (Glostrup, Denmark), respectively. The antibodies to detect DNMT1, VDAC, and total OXPHOS were from Abcam (Cambridge, UK). Anti-p-AKT and PTEN were obtained from Cell Signaling Technologies (Danvers, MA, USA).

Grifolin (2-trans,trans-farnesyl-5-methylresorcinol) was provided by Kunming Institute of Botany, the Chinese Academy of Sciences (purity >99%, High performance liquid chromatography (HPLC) analysis).

Dimethyl sulphoxide (DMSO, Sigma) was used to dissolve grifolin. The final concentration of DMSO in the culture media was kept at <0.1% v/v, which had no significant effect on cell growth.

5-aza-dC (5-aza-2-deoxycytidine) was purchased from Sigma-Aldrich and DMSO was used to dissolve 5-aza-dC.

### Measurement of glucose consumption and lactate production

Cells (5 × 10^5^) were seeded in six-well plates. After incubation for 6 h, culture medium was replaced with fresh medium and incubated for another 8 h. Glucose and lactate levels were measured using the Automatic Biochemistry Analyzer (7170A, Hitachi, Japan).

### GC/MS-based metabolome analysis

Cells were cultured in a glucose-free complete culture medium supplemented with ^13^C_6_-d-glucose at 3.0 g/L for 2 h. Metabolites were then extracted as described previously^70^. Metabolomics instrumental analysis was performed on an Agilent 7890A gas chromatography system coupled to an Agilent 5975C inert MSD system (Agilent Technologies Inc., CA, USA). An OPTIMA^®^ 5 MS Accent fused-silica capillary column (30 m × 0.25 mm × 0.25 μm; MACHEREY-NAGEL, Düren, Germany) was utilized to separate the derivatives. Helium (>99.999%) was used as a carrier gas at a constant flow rate of 1 mL/min through the column. Injection volume was 1 μL in splitless mode, and the solvent delay time was 5.5 min. The initial oven temperature was held at 100 °C for 2 min, ramped to 320 °C at a rate of 10 °C/min and finally held for 8 min. The temperatures of injector, transfer line, and electron impact ion source were set to 250, 250, and 230 °C, respectively. The electron energy was 70 eV, and data were collected in a full scan mode (*m/z* 50–600). The data were analyzed by MSDChem software (Agilent) and corrected using IsoCor software^71^.

### Methylation-specific PCR

Genomic DNA was extracted using QIAamp^®^ DNA Mini Kits (Qiagen) following the protocol established by the manufacturer. The methylation status of *pten* and mitochondrial D-loop region was determined by MSP. Briefly, 2 µg of genomic DNA was modified by the bisulfate reaction using the EZ DNA Methylation-Lightning Kit (Zymo Research, Orange County, CA, USA). Bisulphite-treated DNA was amplified using primers specific for either methylated or unmethylated DNA. The sequences of the methylated-specific (M) primer and unmethylated-specific (UM) primers were: *pten* (U), 5′-GTGTTGGTGGAGGTAGTTGTTT-3′ and 5′-ACCACTTAACTCTAAACCACAACCA-3′; *pten* (M), 5′-TTCGTTCGTCGTCGTATTT-3′ and 5′-GCCGCTTAACTCTAAACCGCAACCG-3′; D-loop (U), 5′-TATAAGAGTGTTATTTTTTTTGTTTT-3′ and 5′-ACATAAATACAAATTATAATAT-3′; D-loop (M), 5′-TATAAGAGTGTTATTTTTTTCGTTTC-3′ and 5′-GCATAAATGCAAATTATAATAT-3′.

### DNMT activity measurement

Freshly isolated nuclear proteins were extracted using the NE-PER Nuclear and Cytoplasmic Extraction Reagents (Thermo Scientific, USA). DNMT1 activity of nuclear extracts was quantified using a fluorimetric EpiQuik™ DNMT Activity Assay Kit (EpigenTek, USA) following the manufacturer’s instruction and calculated as OD/h/mg of protein.

### Isolation of mitochondrial protein fractions

Cells (1.5 × 10^7^) were collected and washed twice with ice-cold 1× phosphate buffered saline (PBS). After centrifugation at 12,000 r.p.m. at 4 °C for 30 min, cell pellets were re-suspended in mitochondrial isolation buffer (70 mM sucrose, 210 mM mannitol, 5.0 mM HEPES, 1.0 mM EGTA, and 0.5% (w/v) BSA, pH 7.2) and then homogenized. Successively, the resulting supernatant fractions were removed and mitochondrial pellets were lysed on ice for 1 h by adding RIPA buffer, protease inhibitor cocktail, and then centrifuged to pellet the mitochondria.

### Immunofluorescence analysis

The cells were grown on glass coverslips overnight and treated with DMSO or grifolin for 5 days, followed by fixation with 4% paraformaldehyde. After permeabilization with 0.3% Triton X-100 in PBS, the cells were blocked with 5% BSA for 1 h and then incubated with the primary antibody (anti-DNMT1) overnight. The coverslips were washed with PBS and then incubated with appropriate fluorescent secondary antibody for 1 h, followed by washing and mounting using DAPI. The images were obtained by confocal microscopy (TCS SP8, Leica) and Pearson’s correlation coefficients for the co-localization of DNMT1 with mitochondria were quantified using the NIH Image J software.

### Measurement of the cellular oxygen consumption rate using the XF extracellular flux analyzer

Cells were plated in XF24 V7 cell culture plates (Seahorse Bioscience, North Billerica, MA, USA) and incubated overnight. Cells were equilibrated with Seahorse XF base medium supplemented with glucose, glutamine, and pyruvate, and incubated into a 37 °C non-CO_2_ incubator for 45 min to 1 h prior to the XF assay. Oligomycin and rotenone were prepared for final concentrations of 1 μM and Carbonyl cyanide-4-(trifluoromethoxy) phenylhydrazone (FCCP) was prepared for a final concentration of 0.5 μM, and were sequentially injected from the reagent ports automatically to the wells. Real-time measurements (triplicate) of oxygen consumption rate (OCR) in pmole per min for cells in culture medium were plotted over time. The OCR measurements were normalized to cell numbers plated.

### Measurement of cellular NADH

NAPH-sensing fluorescence was used to determine cellular NADH level. Cells were seeded into six-well plates and incubated overnight. They were then transfected with the Frex-Mit or cpYFP-Mit plasmid. At 48 h later, the intensity of yellow fluorescence that represented the cellular NADH level was observed by confocal microscopy and quantified using the NIH Image J software.

### CI activity assay

CI activity was determined by measuring the oxidation of NADH to NAD^+^ and the simultaneous reduction of the provided dye (*ε* = 25.9/mM per well), which led to increased absorbance at Optical density (OD) 450 nm. CI activity was quantified using a CI Enzyme Activity Microplate Assay Kit (Abcam) per the manufacturer’s instruction and calculated as mOD/min per µg protein.

### Statistical analysis

All statistical calculations were performed with the SPSS statistical software program (ver.16.0). Differences between various groups were evaluated using a two-tailed Student’s *t* test and a *p* value <0.05 was considered statistically significant.

## Electronic supplementary material

Supplemental Materials and Methods

Supplementary figure 1

Supplementary fig 2

Supplementary fig 3

Supplementary fig 4

Supplementary table 1

Supplementary table 2
