# Supplementary material for: DNMT1 mediates metabolic reprogramming induced by Epstein–Barr virus latent membrane protein 1 and reversed by grifolin in nasopharyngeal carcinoma
Source: Cell Death Dis. 2018 May 23;9(6):619. doi: 10.1038/s41419-018-0662-2 (PMC5966399; doi:10.1038/s41419-018-0662-2)
Supplement: Supplementary file 1 — Supplemental Materials and Methods [file 41419_2018_662_MOESM1_ESM.docx]

**Supplemental Materials and Methods**

**Western blot**

Cells were harvested and washed twice with ice-cold PBS, and then lysed in whole-cell extract buffer (25mM Tris–HCl, pH 7.4, 150mM NaCl, 1%NP40, 1mM EDTA, 5% v/v glycerol). Equal amounts of the total proteins from cell preparations and PageRuler^TM^ molecular weight markers (Fermentas life sciences) were resolved by SDS–polyacrylamide gel electrophoresis and electrotransferred to a PVDF membrane. The membranes were blocked and then incubated with specific primary antibodies according to the manufacturer’s recommendations. The primary antibody complexes were then stained with horseradish peroxidase conjugated secondary antibody and developed with the enhanced chemiluminescence detection kit (ECL;Pierce).

**RNA extraction and quantitative real-time polymerase chain reaction (q-PCR)**

Total RNA was extracted using the TRIzol reagent (Ambion, TX, USA) following the protocol established by the manufacturer. Reverse transcriptional PCR was performed using the RevertAid First Strand cDNA Synthesis kit. The qPCR analysis was performed in a 7500 Real Time PCR System (Applied BioSystems) using the SYBR Green Real-Time PCR kit. The PCR reaction conditions were 10 s at 95 °C followed by 40 cycles of 15 s at 95 °C and 60 s at 60°C.The sequences of primers were:*LMP1 , 5’-CGTTATGAGTGACTGGACTGGA-3’ and5’-TGAACAGCACAATTCCAAGG-3’*

*DNMT1* , 5'-ACCGCCCCTGGCCAAAGCCATTG-3’ and 5'-AGCAGCTTCCTCCTCCTTTATTTTAGCTGAG-3’

*pten,*5'-TCTACTCCTCCAACTCAGGAC-3’ and 5'-CATTATCCGCACGCTCTATAC-3’

*β-actin,*5'-TTCCAGGCCTTCCTTCCTGGG-3’ and 5'-TTGCGCTCAGGAGGAGCAAT-3’.

The *ND6, ATPase6,COXⅡ* genes of the mitochondrial genome was amplified by qPCR using specific primers. Briefly, total DNA was extracted and spectrophotometrically quantitated.200ng DNA templates were then subjected to qPCR. The abundance of mitochondrial target genes was normalized to the amount of *MTRNR2*. The sequences of primers were:*mt-ND6,* 5'-AATAATTTATGAAGGAGAGG-3’ and 5'-CAAACAATGTTCAACCAGTA-3’; *mt-ATPase6*, 5’-CTGAAATCTGTGGAGCAA-3’ and 5’-TATGAGGAGCGTTATGGA-3’; *mt-COXⅡ*，5’-CCATCCCTACGCATCCTTTAC-3’ and 5’-GTTTGCTCCACAGATTTCAGAG-3’; *MTRNR2*,5’-ACTAACCCCTATACCTTCTG-3’ and5’-TTCCCACTATTTTGCTACAT-3’.

**LC-ESI-MS/MS analysis based on Q EXACTIVE HF**

Whole cell lysates were pre-cleared and incubated with mouse anti- OXPHOS antibody. The IP targets were disassociated from the immobilized antibodies on the Dynabeads^TM^ Protein A (Invitrogen). Immunopurified complexes were eluted and resolved on an SDS-PAGE gel, then visualized by coomassie blue staining. The protein band of interest was removed for MS analysis. After protein digestion, each peptide sample was desalted using a Strata X column (Phenomenex), vacuum-dried and then resuspended in a 200 μL volume of buffer A (2% ACN, 0.1%FA). After centrifugation at 20000g for 10min, the supernatant was recovered to obtain a peptide solution with a final concentration of approximately 0.5μg/μl. 10μl supernatant was loaded on a LC-20AD nano HPLC (Shimadzu, Kyoto, Japan) by the autosampler onto a 2cm C18 trap column. Then, the peptides were eluted onto a 10cm analytical C18 column (inner diameter 75μm) packed in-house. The samples were loaded at 8μL/min for 4min, then the 44min gradient was run at 300 nL/min starting from 2 to 35% B (98%ACN, 0.1%FA), followed by 2 min linear gradient to 80%, and maintenance at 80% B for 4 min, and finally return to 5% in 1 min.

The peptides were subjected to nanoelectrospray ionization followed by tandem mass spectrometry (MS/MS) in a Q EXACTIVE HF (ThermoFisher Scientific, San Jose, CA) coupled online to the HPLC. Intact peptides were detected in the Orbitrap at a resolution of 70000. Peptides were selected for MS/MS using high-energy collision dissociation (HCD) operating mode with a normalized collision energy setting of 27.0; ion fragments were detected in the Orbitrap at a resolution of 17500. A data-dependent procedure that alternated between one MS scan followed by 15 MS/MS scans was applied for the 15 most abundant precursor ions above a threshold ion count of 20000 in the MS survey scan with a following Dynamic Exclusion duration of 15 s. The electrospray voltage applied was 1.6 kV. Automatic gain control (AGC) was used to optimize the spectra generated by the orbitrap. The AGC target for full MS was 3e6 and 1e5 forMS2. For MS scans, the m/z scan range was 350 to 2000 Da. For MS2 scans, the m/z scan range was 100-1800. Proteins identification was performed by using Mascot 2.3.02 (Matrix Science, London, UK) against the NCBI database containing 359,414 sequences.

Supplementary figure legends

Supplementary Figure 1 DNMT1 mediates the up-regulation of aerobic glycolysis in LMP1-expressing NPC cells. (A) The mRNA levels of *pten* and *DNMT1* genes in C666-1 cells treated with control siRNA (CON) or 2 different DNMT1 siRNAs (1# and 2#). (B) The protein levels of DNMT1, PTEN and p-AKT were detected by western blot assay in C666-1 cells treated with control siRNA or DNMT1 siRNAs (1# and 2#). (C) Glucose consumption and (D) lactate generation in C666-1 cells treated with control siRNA or DNMT1 siRNAs (1# and 2#). Data are shown as mean values ± S.D. of independent, triplicate experiments. The asterisks (* ,***) indicate significant differences (p < 0.05, p < 0.001,respectively).

Supplementary Figure 2 LMP1 promotes the translocation of DNMT1 to mitochondria, not endoplasmic reticulum. (A) Confocal microscopy images of CNE1 and CNE1-LMP1 cells stained for DNMT1 (green), mitochondria (red), endoplasmic reticulum (purple) and nuclei (blue). Mitochondria were probed by Mitotracker and endoplasmic reticulum by calnexin. Scale bar, 10μm. (B) Pearson’s Correlation coefficients of the co-localization of DNMT1 and mitochondria, or DNMT1 and endoplasmic reticulum were shown by bar graphs. Data are shown as mean values ± S.D. of independent, triplicate experiments. The asterisks (*) indicate significant differences (p < 0.05). NS, not significant (Student’s t-test).

Supplementary Figure 3 Grifolin treatment restores mitochondrial OXPHOS not by targeting LMP1. The OXPHOS complex I-V proteins were detected by western blot analysis in each designated group of (A) C666-1 con and (B) C666-1 shLMP1 cells. (C) Effect of grifolin or 5-aza-dC treatment on the levels of LMP1. CNE1-LMP1 cells were incubated in the basal medium containing 5% serum with DMSO, grifolin (10μM) or 5-aza-dC (10μM) treatment for 5 days, and then subjected to western blot analysis.

Supplementary Figure 4 Effect of grifolin treatment on mitochondrial morphology. (A) CNE1 and (B) CNE1-LMP1 cells were seeded on coverslips overnight and treated with DMSO or grifolin (10 μM) for 5 days, respectively. Mitochondria were stained by Mitotracker and imaged by confocal microscopy. Scale bar, 7.5μm.

Supplementary table 1 Identification of OXPHOS assembly subunits in human nasopharyngeal carcinoma CNE1 cells with or without grifolin treatment

Supplementary table 2 Identification of OXPHOS assembly subunits in human nasopharyngeal carcinoma CNE1-LMP1 cells with or without grifolin treatment

Note: The proteins listed have been identified by using Mascot 2.3.02 (Matrix Science, London, UK) against the NCBI database containing 359,414 sequences. For protein identification, a mass tolerance of 20 Da (ppm) was permitted for intact peptide masses and 0.05 Da for fragmented ions, with allowance for one missed cleavages in the trypsin digests. To reduce the probability of false peptide identification, only peptides with significance scores (≥20) at the 99% confidence interval by a Mascot probability analysis greater than “identity” were counted as identified. Each confident protein identification involves at least one unique peptide.
