# Supplementary figures and images for: DNMT1 mediates metabolic reprogramming induced by Epstein–Barr virus latent membrane protein 1 and reversed by grifolin in nasopharyngeal carcinoma
Source: Cell Death Dis. 2018 May 23;9(6):619. doi: 10.1038/s41419-018-0662-2 (PMC5966399; doi:10.1038/s41419-018-0662-2)

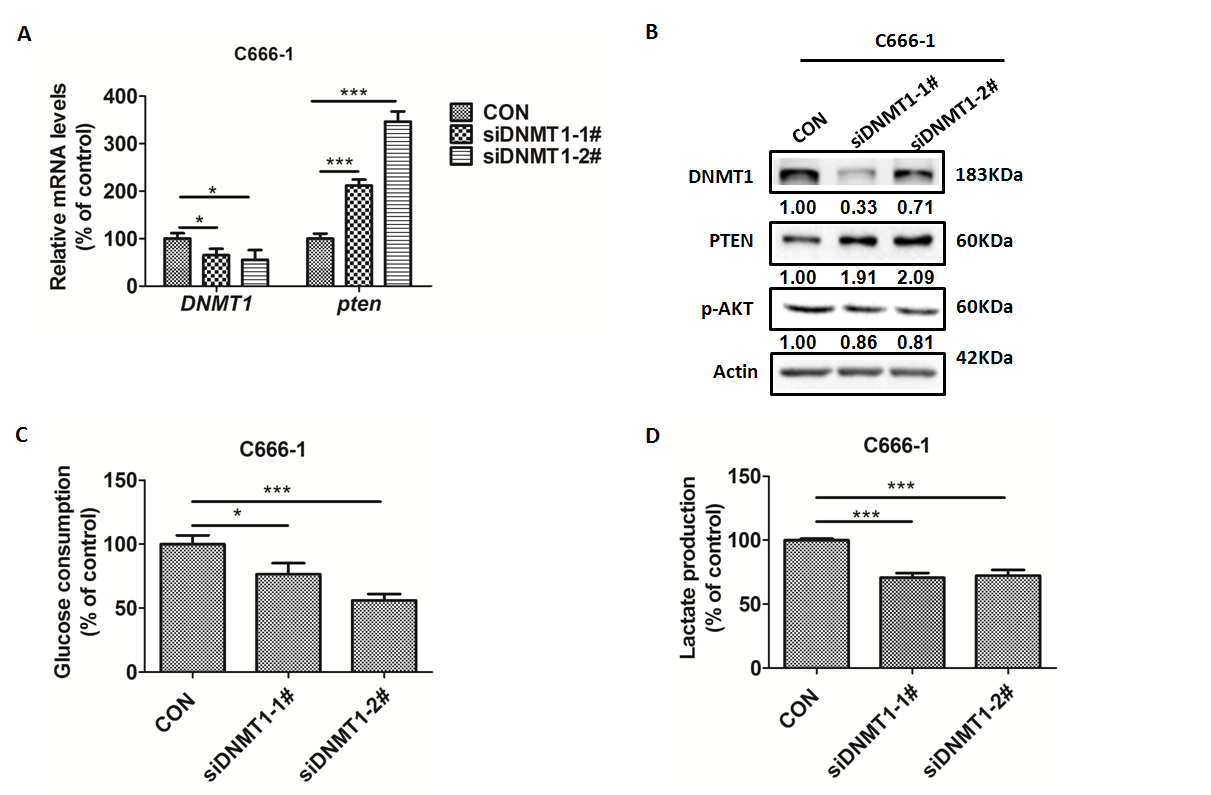

Supplement: Supplementary file 2 — Supplementary figure 1 [file 41419_2018_662_MOESM2_ESM.tif]

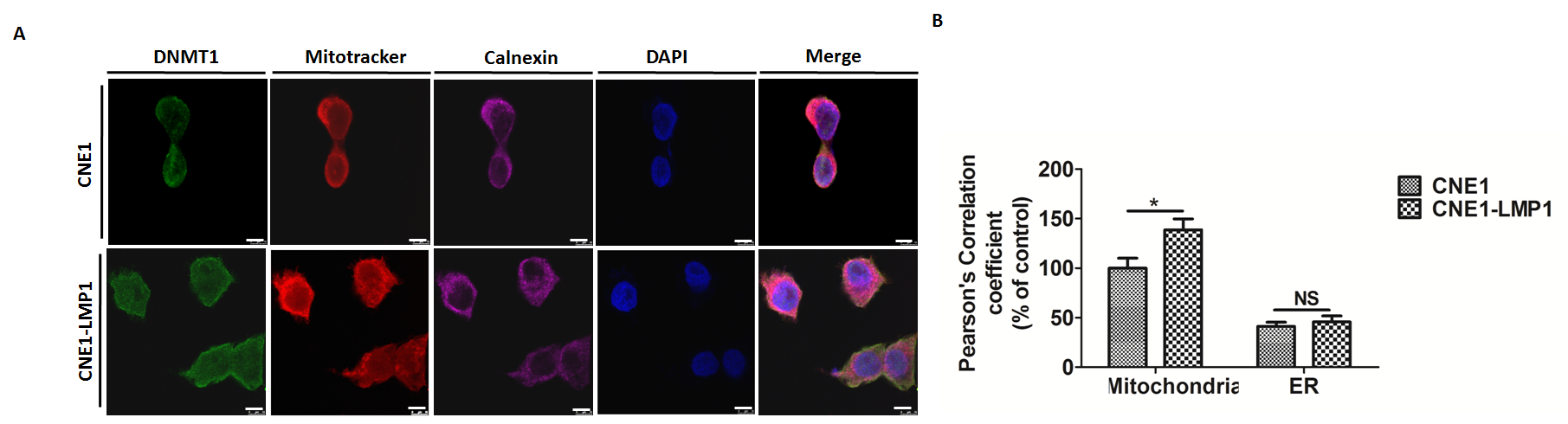

Supplement: Supplementary file 3 — Supplementary fig 2 [file 41419_2018_662_MOESM3_ESM.tif]

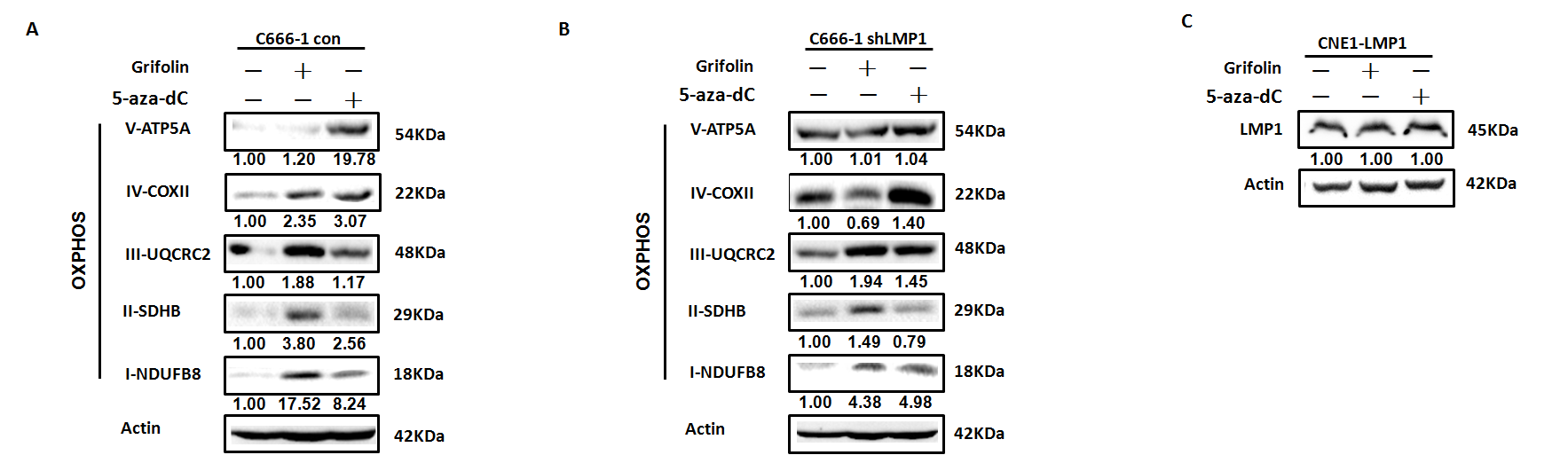

Supplement: Supplementary file 4 — Supplementary fig 3 [file 41419_2018_662_MOESM4_ESM.tif]

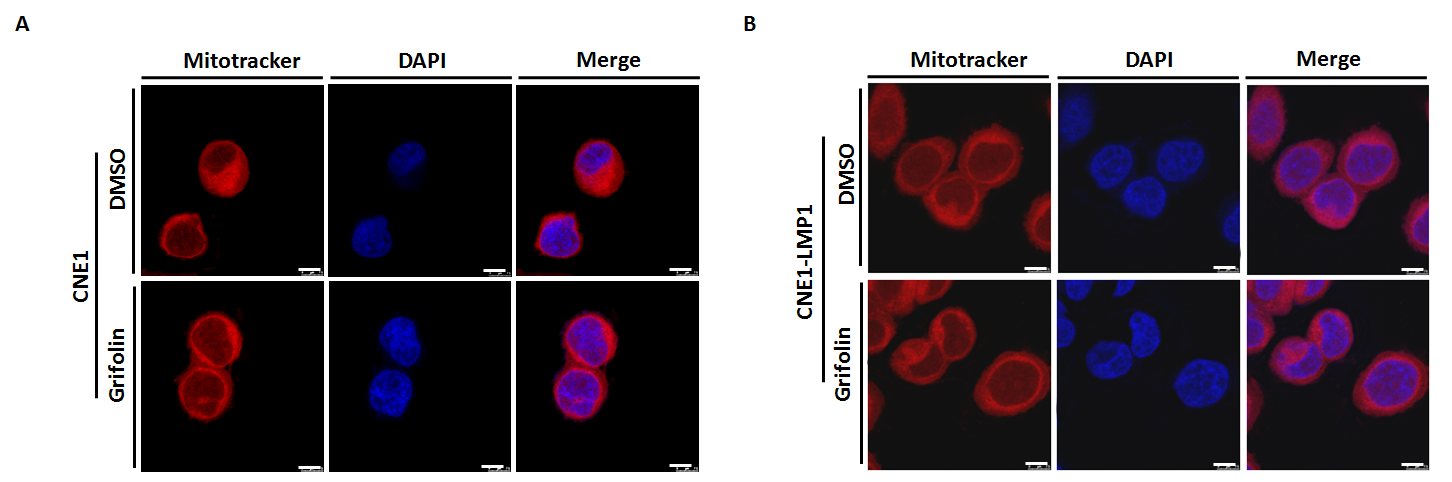

Supplement: Supplementary file 5 — Supplementary fig 4 [file 41419_2018_662_MOESM5_ESM.tif]
